# Progress in the Degradability of Biodegradable Film Materials for Packaging

**DOI:** 10.3390/membranes12050500

**Published:** 2022-05-06

**Authors:** Chuanyan Guo, Hongge Guo

**Affiliations:** School of Light Industry Science and Engineering, Qilu University of Technology, Jinan 250353, China; 10431210995@stu.qlu.edu.cn

**Keywords:** degradable, packaging film materials, degradation mechanism, modified

## Abstract

In today’s world, the problem of “white pollution” is becoming more and more serious, and many countries have paid special attention to this problem, and it has become one of the most important tasks to reduce polymer waste and to protect the environment. Due to the degradability, safety, economy and practicality of biodegradable packaging film materials, biodegradable packaging film materials have become a major trend in the packaging industry to replace traditional packaging film materials, provided that the packaging performance requirements are met. This paper reviews the degradation mechanisms and performance characteristics of biodegradable packaging film materials, such as photodegradation, hydrodegradation, thermo-oxidative degradation and biodegradation, focuses on the research progress of the modification of biodegradable packaging film materials, and summarizes some challenges and bottlenecks of current biodegradable packaging film materials.

## 1. Introduction

Plastic was once hailed as one of the greatest inventions of the 20th century, because of its light weight, good processing performance, low price and many other advantages that make the global plastic industry has been rapid development [1]. According to statistics, the total global production of plastic products exceeds 300 million tons [2,3,4], with 13 million tons entering the water [5]. However, only 6–26% of plastic products are recycled, which means that at least 74% of plastic waste ends up in landfills or enters the environment every year [3,6], of which about 46% comes from the packaging industry, especially food packaging films, which are largely non-recyclable [7]. Since most plastics are now made from non-biodegradable materials, it often takes one to two hundred years to degrade these plastic products [8,9,10,11,12,13].

Plastic is the most commonly used packaging material [14,15], especially packaging film material. However, the packaging industry generates about 141 million tons of plastic waste each year [16], and most of the packaging film materials are composed of non-degradable materials, which obviously leads to many environmental problems, such as “white pollution” [17,18,19]. General purpose plastic packaging films such as polyethylene (PE), polypropylene (PP), polystyrene (PS) and polyvinyl chloride (PVC) [20,21] film materials undergo a long period of aging under the current common waste disposal method of sanitary landfill conditions. Under the action of abiotic factors (such as solar radiation, high temperature, wave impact, gravel abrasion) or biotic factors (such as ingestion, colonization, degradation) [22,23], physical or chemical property changes, molecular weight reduction and molecular weight distribution changes, but its decomposition is not complete, the majority of decomposition into microplastics (particle size < 5 mm) or nanosized-plastics (particle size < 0.1 μm) [24,25]. At present, microplastics have been widely detected in oceans [24,26], sediments [27], rivers [28,29,30], lakes [20], atmosphere [31,32,33], soil [34,35] and organisms [36], disrupting the normal metabolism and energy balance in organisms, thus affecting the normal growth and reproduction of organisms and causing potential harm to human health [37,38].

To solve these problems, it has become important for biodegradable packaging film materials to replace traditional packaging film materials [39,40]. However, biodegradable plastics currently account for less than 1% of total plastics production [41]. Compared with traditional packaging film materials, biodegradable packaging film materials are more expensive to produce and have poor mechanical properties and their barrier properties, which are the main reasons for their limited applications [42].

This paper reviewed the degradation mechanism of different packaging films and the research progress of biodegradable films, and provided outlook on the future development trend of packaging film materials.

## 2. Degradation Mechanism of Degradable Packaging Film Materials

Degraded plastics are plastics that have been subjected to defined environmental conditions for a period of time and contain one or more steps that result in significant changes in the chemical structure of the material resulting in loss of certain properties (such as integrity, molecular mass, structure or mechanical strength) and/or fragmentation [43,44]. As shown in Table 1, the degradation degree can be divided into complete and incomplete degradation, and different degradation mechanisms can be divided into photodegradation, water degradation, thermal oxidative degradation and biodegradation [45].

### 2.1. Photodegradation

Photodegradable materials are degraded to low molecular weight compounds that are relatively safe for the environment by photo-initiated fracture and free radical oxidative fracture reactions under the action of sunlight (mainly UV light) [46]. Photodegradable film materials can be mainly divided into photodegradable materials obtained by copolymerization and photodegradable materials with composite photosensitizers [47].

**Figure 1:**
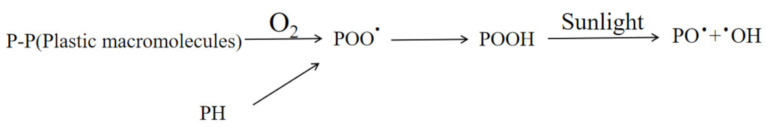
The mechanism of photodegradation.

In sunlight, UV light with a wavelength of 290 nm–400 nm only accounts for about 5%, and it is the UV light that causes photodegradation of the film. Figure 1 shows the photodegradation mechanism. The molecular chains react under certain conditions of oxygen, temperature and humidity, and the long molecular chains are decomposed into peroxides and eventually achieve photodegradation [48].

Christensen et al. [49] investigated the photodegradation properties of polymers with a 1:1 mass ratio of polycaprolactone to polyvinyl chloride by monitoring CO_2_ emissions during UV exposure. The results showed that the interaction of the two components in the polymer reduced the photodegradability. Najaf et al. [50] used polyaniline modified TiO_2_ as a photocatalyst and then combined it with polyvinyl chloride to make photodegradable films. The results showed that the quality of polyaniline decreased by 67% when the molar ratio of polyaniline to TiO_2_ was 10:1 under the condition of 30W UV lamp irradiation for 720 h, decreased by 12% compared with the pure polyvinyl chloride (PVC) film, and its photodegradation performance was greatly improved.

Photodegradable materials must be exposed to light and have a long degradation period, while most film materials are not exposed to natural light for a long time after disposal and it is difficult to ensure the degradation conditions required for photodegradable film materials, which greatly limits the large-scale application of photodegradable film materials.

### 2.2. Hydrodegradation

Hydrodegradable plastic is a kind of plastic that can self-degrade by hydrolysis. The essence is the presence of hydrolyzable covalent bonds in degradable plastics, such as esters, ethers, anhydrides, amides, carbamides or ester-amide groups [45], which can achieve dissolution when the plastic encounters water [51,52]. Water activity, temperature, pH and time are the key factors affecting the efficiency of hydrolysis [53].

Polyvinyl alcohol (PVA) is a water-soluble polymer with a carbon chain as the main chain and a large number of hydroxyl groups on the side chain [54,55]. It is non-toxic, easily processed, biodegradable, has good mechanical properties [56,57], and can be mixed with natural polymeric materials such as polysaccharides and proteins to improve its properties [58,59,60]. Mainly used in the packaging of water-soluble products, the buyer can do not touch the product in the process of using the product, safe and at the same time make the use of the product more convenient. However, the resistance of PVA film to water is very low, usually in a very short period of time can be completely dissolved [61]; therefore, if it is widely used in the field of packaging needs, it needs to be modified for water resistance.

Lv et al. [62] investigated the time-dependent hydrolysis behavior of polylactic acid (PLA) and starch/PLA composites. The results showed that the presence of starch may induce hydrolysis to occur at the interface between starch and PLA. In addition, starch can slightly slow down PLA hydrolysis without affecting the degree of PLA hydrolysis. Table 2 shows the water degradation of several common biodegradable polyesters in different water environments.

**Table 2:** Hydrologic degradation of several typical biodegradable polyesters in different water environments. Data from [63].

Material	Conditions	Weight Loss %	Number-Average Molecular Weight (Mn)	Mechanical Properties
Polylactic acid (PLA)	Seawater	<2	96.60 × 10^3^ to 83.85 × 10^3^	No significant change
	Germicidal water	<2	96.60 × 10^3^ to 67.98 × 10^3^	
Poly (butyleneadipate-co-terephthalate) (PBAT)	Seawater	<2	46.67 × 10^3^ to 20.31 × 10^3^	Total loss
	Germicidal water	<2	46.67 × 10^3^ to 16.02 × 10^3^	
Poly (butylene succinate) (PBS)	Seawater	<2	41.56 × 10^3^ to 30.11 × 10^3^	Total loss
	Germicidal water	<2	41.56 × 10^3^ to 18.63 × 10^3^	
Polycaprolactone (PCL)	Seawater	32	77.79 × 10^3^ to 77.09 × 10^3^	Total loss
	Germicidal water	<2	77.79 × 10^3^ to 14.82 × 10^3^	

### 2.3. Thermal Oxidative Degradation

**Figure 2:**
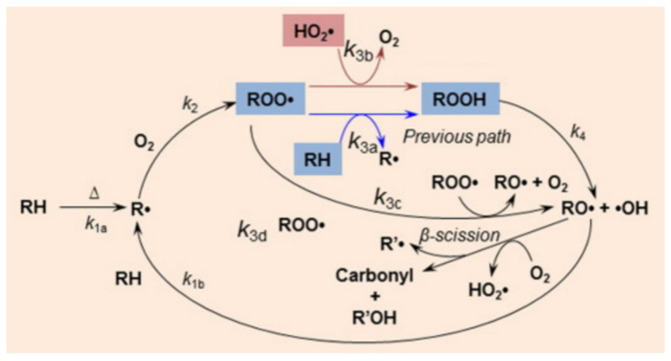
Auto-oxidation scheme of polymer. Reprinted from Ref. [68]. Copyright (2016), with permission from Elsevier.

Thermally oxygen degraded plastic is that subjected to heat and/or oxidation over a period of time and contains one or more steps that result in significant changes in the chemical structure of the material, resulting in loss of certain properties (such as integrity, molecular mass, structure or mechanical strength) and/or fragmentation [64,65]. Heat can change the oxidation mechanism of plastics, and higher temperatures can improve the degradation of plastics [66,67]. Figure 2 shows the mechanism of thermal oxidative degradation. Thermally oxygen degraded plastic is also very difficult to degrade completely in most cases due to the conditions.

Gaurav et al. [69] prepared two high-density polyethylene/polylactic acid blends with and without the addition of a compatibilizer and a pro-oxidant using a melt blending technique. The results showed that the addition of the compatibilizer led to a significant improvement in the mechanical properties of the blends and the addition of the pro-oxidant led to an improvement in their oxidative degradation properties.

### 2.4. Biodegradable

**Figure 3:**
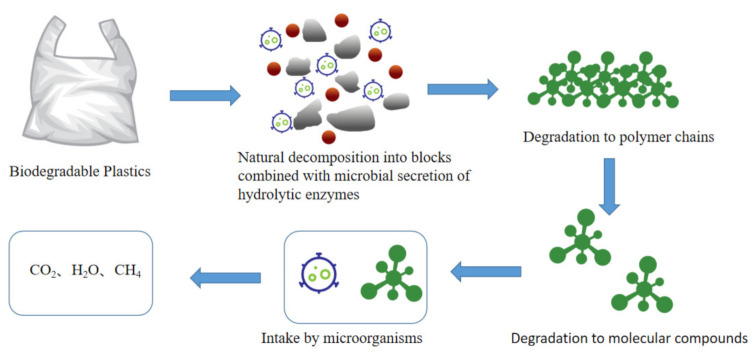
The mechanism of biodegradation.

Biodegradable plastics are those degraded by naturally occurring microorganisms under natural conditions such as soil and/or sand, and/or specific conditions such as composting or anaerobic digestion or aqueous cultures, and ultimately degrade to environmentally benign biomass, CO_2_, CH_4_ and H_2_O [70,71,72]. Figure 3 shows the biodegradation mechanism. Biodegradable plastics have stable performance and can be completely degraded and returned to nature in a short period of time under composting conditions [73].

Current research shows that animals, plants, microorganisms and enzymes all have some ability to degrade plastics [74,75]. Table 3 shows the biodegradation of common plastics. Among the many ways to change the properties of plastics, biodegradation of plastics is one of the inevitable environmental processes for plastics to enter the environment, and it is also an in situ, green, relatively low-cost and low-technology way to treat plastic waste.

**Table 3:** Biodegradation of common plastics.

Material	Conditions	The Result of Degradation	References
Polyethylene	Degradation of high-density polyethylene with *Aspergillus flavus* PEDX3 strain for 28 days	Molecular weight reduction	[76]
Polypropylene	Degradation of polypropylene with microalgae *Spirulina* sp. for 112 days	Decrease in mechanical strength and relative molecular weight	[77]
Polystyrene	Degradation of polystyrene with *Achatina fulica* for 4 weeks	The mass loss was 30.7% on average, forming a functional group of oxidation intermediates	[78]
Polyethylene terephthalate	Degradation of polyethylene terephthalate with microalgae *Spirulina* sp. for 112 days	Decrease in mechanical strength	[77]
Polylactic acid	Degradation in accordance with ISO 17556	15% of Polylactic acid is degraded	[79]

Among various degradable mechanisms, biodegradation is more complete and faster than other degradation mechanisms, and the degradation products are harmless. Biodegradable plastics can be composted together with organic waste, thus eliminating the manual sorting step compared to general plastic waste, greatly facilitating waste collection and disposal, thus making composting and harmless disposal of organic waste into reality [80]. Biodegradable packaging film materials are green, environment-friendly and resource-saving compared with traditional film materials, thus gradually becoming a research hotspot in the packaging industry, the development of biodegradable packaging film is an effective way to fundamentally solve “white pollution”.

## 3. Biodegradable Film Materials

Biodegradable film materials can be divided into three categories according to raw materials and processing methods: natural polymer-based films, petroleum-based film materials and bio-based film materials.

### 3.1. Natural Polymer-Based Films

#### 3.1.1. Starch-Based Film Materials

Starch is a natural degradable polymer [81], available from a wide range of plant species [82], the long chain molecules can be broken into glucose monosaccharides and other small molecules by the action of microorganisms, and eventually metabolized to CO_2_ and H_2_O [83]. Starch-based films are one of the most productive biodegradable films in the world, with high flexibility, good oxygen barrier, colorless, environmentally friendly and other characteristics [84], but its film also has a difficult to process, physical properties and poor water resistance as well as other disadvantages [85], so in practical use, they usually need to be modified [86]. Surface modification [87,88], blending with reinforcement fillers [89,90,91], and blending with other polymers [92,93] are the three most commonly applied reinforcement strategies. Table 4 shows the different modifications of starch-based film materials.

**Table 4:** The different modifications of starch-based film materials.

Modification	Conditions	Result	References
Blending with other polymers	Modified starch-based film materials with natural fibers in blends	Tensile strength and modulus of elasticity were improved, but the elongation at break was not as good as that of ordinary starch-based films	[92]
Blending with other polymers	Modified barley hulls (BH) by grafting palmitic acid and then blended with cross-linked polyvinyl alcohol (PVA)/starch	The physical properties of the composite film could be effectively improved, and the air and water resistance were substantially enhanced	[93]
Surface modification	Acetylated corn starch (AS), acetylated sugarcane fiber (AcSF) and glycerol were used to make biodegradable film materials	Mechanical properties and water resistance have been improved	[87]
Blending with reinforcement fillers	Different contents of metakaolin were blended with cassava starch to make film materials	The mechanical tensile strength and properties increased significantly and the elongation at break decreased	[91]

#### 3.1.2. Cellulose-Based Film Materials

Cellulose is a highly reactive biopolymer with a large amount of hydroxyl group in its structure [94,95,96,97], which can be chemically modified through various reactions such as esterification, etherification and oxidation to give cellulose new properties while ensuring its degradable properties [98,99]. As a packaging material, cellulose also has good biodegradability and excellent physical and mechanical properties, which makes it one of the most suitable natural polymers for use in films [98,100]. However, cellulose also has some limitations, such as high water absorption and poor interfacial adhesion [101]. Cellulose is also converted into various derivatives, which can be mainly classified as: cellulose acetate (CA), cellulose sulfate (CS), cellulose nitrate (CN), carboxymethyl cellulose (CMC), ethyl cellulose (EC), methyl cellulose (MC), cellulose nanocrystals (CNC) and nanocellulose (NC) [98]. Table 5 shows examples of cellulose derivatives film formation.

**Table 5:** Examples of cellulose derivatives film formation.

Material	Conditions	The Result of Degradation	References
Cellulose acetate (CA)	The film material was produced by mixing CA, sodium alginate (SA) and carrageenan (CG) by solution casting method	The tensile strength, thermal stability and antimicrobial activity of the films were improved	[102]
Nanocellulose (NC)	Nanocellulose is used as filler for melt blending and blown film with PLA	The mechanical strength, crystallinity and wettability are improved	[103]
Cellulose nanocrystals (CNC)/ Carboxymethyl cellulose (CMC)	CMC films containing various contents of CNC were prepared by solution casting method	Compared with pure CMC films, CMC/CNC composite films have better UV barrier, mechanical strength, water vapor barrier and thermal stability	[104]
Ethyl cellulose (EC)	Preparation of PVA/EC/tea polyphenol (TP) nanofiber films by blending electrospinning technique	The thermal stability, surface hydrophobicity, water resistance, water vapor barrier capacity and tensile properties of the composite nanofiber films were improved	[105]

#### 3.1.3. Chitosan-Based Film Materials

As the second most abundant polysaccharide in nature after cellulose [106,107], chitosan (CS) is not only widely available and biodegradable, but also has good film-forming, biocompatible and antibacterial properties [108,109], and is one of the ideal materials for packaging films. However, its poor mechanical properties, weak water resistance and poor thermal stability also limit the application in packaging [110,111]. Properties can be improved by cross-linking [112,113], graft copolymerization [114,115], blending with reinforcement fillers [116] and blending with other polymers [117]. Table 6 shows the different modifications of chitosan-based film materials.

**Table 6:** The different modifications of Chitosan-based film materials.

Modification	Conditions	Result	References
Cross-linking	Preparation of a chitosan/bacterial cellulose membrane treated by multiple cross-linking methods	Mechanical strength and elongation at break increase, but its antimicrobial efficiency decreases	[112]
Graft copolymerization	Chitosan (CS) was grafted with caffeic acid (CA-g-CS) through carbodiimide coupling and cast into films	CA-g-CS films have higher tensile strength, elongation at break and oxidation activity, and better barrier properties to water vapor and oxygen	[114]
Blending with reinforcement fillers	Nickel oxide nanoparticles (NiONPs) were doped into chitosan-based films to fabricate composite films	The composite film has improved water resistance, tensile strength, thermal properties and surface hydrophobicity, and has ideal photocatalytic and antibacterial activity	[116]
Blending with other polymers	Biodegradable chitosan-based film containing micro ramie fiber and lignin was prepared by the casting method	Significant improvement in mechanical, water resistance, thermal and oxidation resistance properties	[117]

### 3.2. Petroleum-Based Film Materials

#### 3.2.1. Poly (Butylene Succinate) Film Materials

Poly (butylene succinate) (PBS or PBSu), an aliphatic polyester, can be contained in petrochemical-based biodegradable polymers [118,119], but the important novelty is that PBS can be produced from renewable resources such as sugarcane, cassava and corn [120,121]. PBS has similar properties to PE, so it is often compared to PE and appears as a biodegradable alternative [122]. PBS has excellent properties, such as elongation at break of over 200% and good barrier properties [7,123]. However, the relatively high cost still limits its application. Therefore, many strategies are being developed to reduce costs, on the one hand, and improve its performance to meet the specific requirements of packaging, on the other. Blending with other polymers [124,125,126,127], synthetic copolymers [128,129] and blending with reinforcement fillers [130,131] are commonly used to improve their properties. Table 7 shows the different modifications of PBS film materials.

**Table 7:** The different modifications of PBS film materials.

Modification	Conditions	Result	References
Blending with other polymers	The PBS and plasticized whey protein (PWP) blend makes the film	Significant increase in modulus of elasticity, tensile strength and elongation at break	[124]
Blending with other polymers	Preparation of PCL/PBS co-blended film by immersion precipitation	Improved hydrophilicity and biodegradability, in addition to higher pollution inhibition index	[127]
Synthetic copolymers	Synthetic poly (butylene succinate-co-diethylene glycol succinate) (P(BS-co-DEGS)) copolymer	Crystallinity, tensile modulus, thermal stability slightly reduced and water degradation rate increased.	[129]
Blending with reinforcement fillers	Preparation of PBS/graphene nanoplatelets (GnP) nanocomposites	Improved barrier properties to water and oxygen	[131]

#### 3.2.2. Poly (Butyleneadipate-co-Terephthalate) Film Materials

Poly (butyleneadipate-co-terephthalate) (PBAT) is an aliphatic-aromatic copolyester mainly made by condensation of benzodimethyl (C_8_H_6_O_4_), adipic acid (C_6_H_10_O_4_) and butylene glycol (C_4_H_10_O_2_), etc. [132,133,134]. In addition to biodegradability, PBAT has high flexibility, high strength and good tear resistance, and is widely used in various industries, especially in food packaging [135,136]. Pure PBAT films have higher costs and lower mechanical properties than traditional film materials [133,137,138], so blending with reinforcement fillers [139,140,141,142] or blending with other polymers [134,143] is an effective way to reduce prices and improve performance. Table 8 shows the different modifications of PBAT film materials.

**Table 8:** The different modifications of PBAT film materials.

Modification	Conditions	Result	References
Blending with reinforcement fillers	Starch/PBAT nanocomposite films with high starch content were prepared by extrusion blow molding	Significant increase in mechanical strength, flexibility and hydrophobicity	[141]
Blending with reinforcement fillers	Preparation of PBAT/lignin composite films by extrusion hot-pressing	Significantly improved flexibility and mechanical properties	[140]
Blending with other polymers	Compression molded biodegradable films based on PBS and PBAT at varying weights were prepared	Elongation at break increased with increasing PBAT content, and gas barrier properties decreased with increasing PBS content.	[143]
Blending with reinforcement fillers	Preparation of PBAT/TiO_2_ biodegradable films	The addition of TiO_2_ leads to the improvement of the overall barrier properties, thermal stability and tensile strength of PBAT composite film materials, but its elongation at break decreases	[142]

#### 3.2.3. Polycaprolactone Film Materials

Polycaprolactone (PCL) is a green, non-toxic synthetic aliphatic polyester material [144] with numerous advantages, including: (1) faster crystallization rate and higher crystallization [145]; (2) rubbery state at room temperature, elongation at break hundreds of times higher than PLA [146,147]; (3) better rheology, viscoelasticity, good flexibility and processability [148]; (4) outstanding resistance to UV radiation, wear resistance, anti-aging properties, longer degradation half-life than PLA [127,149]; (5) excellent biocompatible and biodegradable, non-toxic and harmless, EU and FDA certified for implantation into human body [150]; (6) strong hydrophobicity and drug passage [151]. However, it has the characteristics of poor water solubility, slow degradation, low melting point and poor mechanical strength, so it needs to be modified in the actual use process. Table 9 shows the different modifications of PCL film materials.

**Table 9:** The different modifications of PCL film materials.

Modification	Conditions	Result	References
Cross-linking	Polycaprolactone (PCL) was cross-linked by adding different amounts of organic peroxides, such as di-(2-tert-butylperoxyisopropyl)-benzene (BIB)	PCL branching and cross-linking have significant effects on the mechanical properties of PCL 0.5 pbw (part by weight) BIB-modified PCL has better mechanical properties, and higher BIB content can lead to degradation and excessive cross-linking of PCL	[152]
Compound modification	Prepared PCL/polyvinyl chloride (PVC)/organoclay nanobioblends film	Enhanced mechanical and barrier properties, exhibiting some antibacterial activity	[153]
Blending with other polymers	PCL/PLA is mixed and green tea extract (GTE) is used as an antioxidant to make the film	Reduced hydrophilicity and enhanced barrier and mechanical properties	[154]

### 3.3. Bio-Based Film Materials

#### 3.3.1. Polyhydroxyalkanoates Film Materials

Polyhydroxyalkanoate (PHA) is a general term for a class of biopolyester produced by microbial fermentation engineering technology, which has good biocompatibility and biodegradability [155,156], and has the thermoplastic processability of petrochemical resins, which can be processed by injection molding, extrusion blow molding film, extrusion calendering, extrusion hollow molding, compression molding, etc., and manufactured into films and containers that are widely used in packaging [157,158]. PHAs are classified into short chain length and medium chain length PHAs, which depend on the amount of carbon in the monomeric fraction [159,160]. Its main varieties are poly-β-hydroxybutyric acid (PHB), poly-β-hydroxyvalerate (PHV) and their copolymers poly (β-hydroxybutyrate-β-hydroxyvalerate) (PHBV), etc. [161]. Besides having some advantages, it is limited by poor mechanical properties, high susceptibility to thermal degradation and high production cost in practical applications [162], so it needs to be modified. Table 10 shows the different modifications of PHA film materials.

**Table 10:** The different modifications of PHA film materials.

Modification	Conditions	Result	References
Copolymerization modification	Four cross-linkers (citric acid, adipic acid, borax and boric acid) with polycarboxyl or polyhydroxy structures were used in the preparation of the starch/polyhydroxyalkanoate (PHA) films	With higher relative crystallinity, but hinders the formation of intercalation structures in the polymer matrix, improving light transmission and barrier properties	[163]
Blending with reinforcement fillers	Lignin nanoparticles homogeneously dispersed in poly-β-hydroxybutyric acid (PHB) matrix to form nanocomposites with improved properties using oil-in-water emulsion method	Improved mechanical properties, lower crystallinity, higher glass transition temperature and better barrier properties	[164]
Compound modification	Preparation of PHA/PLA nanocomposite films under different levels of montmorillonite	Better thermal stability and electrical conductivity	[165]

#### 3.3.2. Polylactic Acid Film Materials

Polylactic acid (PLA) is a type of degradable polymer material with lactic acid as raw material [166,167], which is renewable and has the characteristics of non-toxicity, non-irritation, good biocompatibility, processability, excellent mechanical properties, complete biodegradability and environmental friendliness [168,169], and is considered as the main alternative to petroleum-based plastics [170].

**Figure 4:**
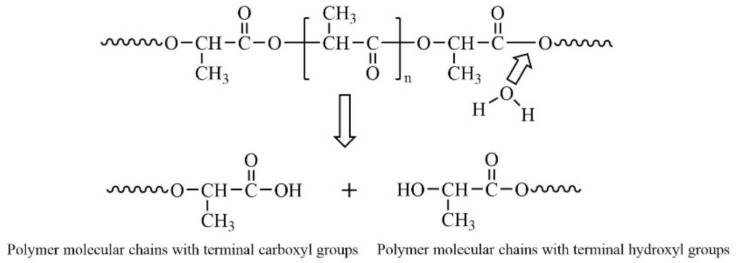
Hydrolysis mechanism of PLA. Reprinted from Ref. [175]. Copyright (2016), with permission from Springer.

The degradation of PLA in nature occurs first by water degradation and then by biodegradation, and the hydrolysis of PLA films is mainly caused by the hydrolysis of the ester bond of the main chain into molecules of low relative molecular mass [171,172,173,174], and the hydrolysis process is shown in Figure 4 [175].

The biodegradation of PLA is greatly influenced by environmental factors [176]. The start of hydrolysis at room temperature takes six months, while biodegradation takes one year, and microorganisms play almost no role in the beginning of hydrolysis, which is one of the characteristics of PLA [176,177].

PLA is one of several biodegradable plastics with large global production, PLA has sufficient raw material sources, is renewable and has good hardness, gloss and thermoplasticity, as well as good tensile strength and processing properties, but it also has deficiencies such as poor blending properties and expensive [178]. Currently, the comprehensive performance of PLA is mainly improved by copolymerization modification, blending with reinforcement fillers, blending with other polymers and compound modification [179,180,181]. Table 11 shows the different modifications of PLA film materials.

**Table 11:** The different modifications of PLA film materials.

Modification	Conditions	Result	References
Blending with reinforcement fillers	Add bamboo cellulose nanowhiskers (BCNW) to PLA as a filler and make a film by solution casting method	Mechanical properties, glass transition temperature, cold crystallinity increase and microcrystal size increase significantly	[180]
Compound modification	Introduction of glass fibers (GF) modified with silane coupling agent (GF-S) into PLA to make PLA-based composites	Improved mechanical and thermodynamic properties	[179]
Blending with reinforcement fillers	Halloysite nanotubes (HNT) and chitosan as fillers were blended with PLA to make films	Mechanical strength and mechanical properties have been improved, with excellent barrier to water and UV light, and some antibacterial ability	[166]
Blending with other polymers	Cinnamic acid (CA)/PLA films obtained by casting or thermal processing	Greatly improves the mechanical properties of the film and improves the barrier to oxygen and water	[182]
copolymerization modification	PLA is blended with polydecalactone (PDL)-grafted cellulose copolymer (CgPD) and made into films	Improved mechanical properties and mechanical properties	[183]

PLA film is the most cost-effective biodegradable film material. Its mechanical properties and transparency are similar to those of polystyrene (PS) or polyethylene terephthalate (PET) films, and it has good processability and stable performance, making it a promising biodegradable film. Compared with other materials, there are many PLA materials that are already used in business. R. J. Reynolds Tobacco Products (US) has developed a new tobacco packaging film coated with a metal oxide layer consisting of aluminum oxide, titanium oxide and/or aluminum-titanium oxide that retains some moisture barrier capability at the fold after folding [184]. Suzhou Xinghuo Fengying Environment Prot Package Co Ltd. has developed a blown film process using PLA, PBAT, antioxidants and poly (N-propionylethylenimine) blended extrusion, according to which the garbage bags produced by this process have high elongation at break [185]. Perak Biochemicals has developed a method for producing polylactic acid (PLA)-shaped products by thermoforming and such thermoformed PLA products. Purac Biochem Bv (NL) Perak Biochemicals has developed a method for producing polylactic acid (PLA)-shaped products by thermoforming and such thermoformed PLA products [186].

## 4. Summary and Outlook

This review focuses on the degradation mechanism of packaging films and the properties and performance of several common biodegradable film materials available today. This includes natural polymer-based film materials, petroleum-based film materials and bio-based film materials. With the global “plastic ban”, the development of biodegradable packaging films is one of the important research directions to solve the resource and environmental problems. However, biodegradable packaging films also have poorer performance than traditional packaging films, insufficient degradation controllability and higher production costs. How to reduce the material production cost by improving the synthesis and process is an urgent issue for the massive use of biodegradable films. In response to the disadvantages of poor performance of biodegradable films, the development of modification technologies such as cross-linking modification, hybrid modification, copolymer modification and composite modification has become one of the key research directions at present. The degradation performance of biodegradable packaging film is also an important factor affecting its use, and either too fast or too slow degradation will limit its use. To master the degradation mechanism of biodegradable packaging films and then realize the controlled degradation of packaging films is a challenge that needs to be overcome by the efforts of researchers.

At present, the misuse of biodegradable film materials in the market places a great burden on the environment. Since they are expensive, some manufacturers usually add some petroleum-based plastics to biodegradable products to improve durability as well as reduce cost. However, this can lead to “pseudo-degradation” and can result in microplastics entering the environment, causing a greater burden on the environment. Therefore, developing biodegradable standards for the packaging industry will also be a priority. The global outbreak of the coronavirus disease (COVID-19) in 2019 has gradually started to bring antibacterial and antiviral packaging films into the limelight, and food safety issues have become too important to ignore, and the development of antibacterial and antiviral biodegradable packaging films will be an important research direction. Research and development of biodegradable packaging films with better performance, economy and convenience is the main task of the packaging industry all over the world, replacing traditional packaging films with biodegradable packaging films to achieve green development in the packaging field.

## Figures and Tables

**Table 1:** The classification and characteristic of degradable plastics.

Classification	Category	Features
By degradation principle	Biodegradable plastics	Similar performance to traditional plastics, good degradability, high safety
	Photodegradable plastics	Simple and low cost production process
	Thermal oxidative degradation plastics	Requires oxygen and heat
	Hydrodegradable plastics	Short degradation time, no trace, no pollution, low cost
By degradation characteristics	Fully degradable plastics	Completely disintegrates and leaves no trace
	Incomplete degradable plastics	Partial degradation

## Data Availability

Not applicable.
